# Increased resting lactate levels and reduced carbohydrate intake cause νLa.max underestimation by reducing net lactate accumulation—A pilot study in young adults

**DOI:** 10.14814/phy2.70020

**Published:** 2024-08-26

**Authors:** Alexander Pohl, Frederik Schünemann, Kirill Schaaf, Woo‐Hwi Yang, Hermann Heck, Oliver Heine, Daniel Jacko, Sebastian Gehlert

**Affiliations:** ^1^ Department for Biosciences of Sports Institute of Sport Science, University of Hildesheim Hildesheim Germany; ^2^ Institute of Cardiovascular Research and Sports Medicine, German Sport University Cologne Cologne Germany; ^3^ Olympic Base Center, North Rhine‐Westphalia/Rhineland Cologne Germany; ^4^ Graduate School of Sports Medicine CHA University Pocheon Republic of Korea; ^5^ Faculty for Sports Sciences Ruhr Universität Bochum Bochum Germany

**Keywords:** carbohydrate intake, exercise performance, glycolytic metabolism, maximal glycolytic rate, net lactate accumulation, νLa.max

## Abstract

Modulation of testing conditions such as resting lactate (La_rest_) levels or carbohydrate intake may affect the calculation of the maximal glycolytic rate (νLa.max). To evaluate the impact of elevated La_rest_ as well as reduced and increased carbohydrate availability on νLa.max in running sprints (RST), twenty‐one participants completed five 15‐s RST tests on a running track under five different conditions: (I). baseline: La_rest_ ≤1.5 mmol·L^−1^; (II). Lactate+: La_rest_ ≥2.5 mmol·L^−1^; (III). CHO*−*: carbohydrate intake: ≤ 1 g·kg^−1^ BW d^−1^ for 3 days; (IV). CHO+: carbohydrate intake: ≥ 9 g·kg^−1^ BW d^−1^ for one day; and (V). acuteCHO: 500 mL glucose containing beverage consumed before RST. νLa.max was significantly reduced in lactate+ and CHO− conditions compared to the baseline RST, due to a reduction in the arithmetic mean delta (∆) between La_peak_ and La_rest_ lactate concentration (La_peak_, mmol · L^−1^). AcuteCHO led to an increase in La_rest_ compared to baseline, CHO− and CHO+ with a high interindividual variability but did not significantly reduce νLa.max. Therefore, avoiding low carbohydrate nutrition before νLa.max testing, along with carefully adjusting La_rest_ to below ≤1.5 mmol·L‐1, is crucial to prevent the unintentional underestimation of νLa.max.

## Graphical abstract

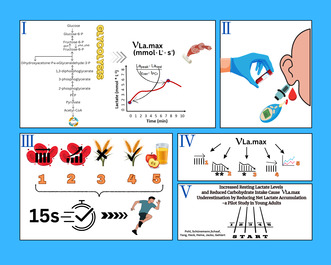

## 1 INTRODUCTION

Originally introduced in 1984 by Alois Mader (Mader, 1984), the maximal glycolytic rate (νLa.max) has recently resurrected as a surrogate of anaerobic glycolytic exercise performance (Langley et al., 2024; Park et al., 2021; Quittmann, Schwarz, et al., 2021; Yang et al., 2023). Meanwhile νLa.max also gained popularity in the field of athletic testing, as it is frequently discussed in various online forums (Xxxx, 2024a, 2024b, 2024c).

It has been proposed that νLa.max influences endurance performance because a higher νLa.max results in a lower power output at the maximum lactate steady state (MLSS) and is associated with higher lactate concentrations at any workload compared to an athlete with the same V̇O_2max_ but lower νLa.max (Wackerhage et al., 2022). In contrast, some findings attribute a higher glycolytic performance as important for sports disciplines where high intense muscle contractions have to be carried out at workloads above MLSS (Wackerhage et al., 2022) or also V̇O_2max_ (Heck et al., 2003; Wackerhage et al., 2022). It has been shown that νLa.max correlates negatively with percentual usage of VO_2max_ and positively with the ratio of the final 200 m of a 5000 m run (*finishing kick*) (Quittmann et al., 2023) in track athletes. In rowing, the glycolytic energy contribution is well associated with νLa.max (Schünemann et al., 2023). Here, the power of the first 300 m of a 2000 m rowing time trial and the Δ300first‐last were shown to be positively correlated with the rowing specific νLa.max (Schünemann et al., 2023). Additionally, the νLa.max determined in swim sprints over 25 m, 35 m, and 50 m correlate with the swimming speed. Such findings implicate that the νLa.max contributes to sport specific performance (Mavroudi et al., 2023) provided that it can be reliably measured in appropriate testing procedures. On a scientific basis νLa.max has recently been assigned as being reliable (Held et al., 2024) and a growing number of scientific publications treat methodological aspects of νLa.max determination (Andrade et al., 2015; Keir et al., 2013; Meckel et al., 2009; Quittmann, Abel, et al., 2021; Quittmann et al., 2020).

The determination of νLa.max is initiated by a maximal sprint performance during which skeletal muscle glycolysis is maximally stimulated and the produced skeletal muscle lactate is then released into the blood stream. νLa.max is subsequently determined as the difference between resting blood lactate (La_rest_) and the maximally accumulated post‐exercise blood lactate (La_peak_) (Heck et al., 2003). This numerator is then divided by an alactic time which is subtracted from the entire time of the sprint (see Equation 1) (Heck et al., 2003). Methodologically, La_rest_ can be manipulated by any physical activity beforehand a νLa.max test or short resting times between a warmup procedure and the νLa.max test (Beneke et al., 2011). As lactate is produced via glucose or glycogen in the anaerobic glycolysis, acute or chronic changes in carbohydrate availability status can influence La_rest_ (Hu et al., 2020) and La_peak_ (Gollnick et al., 1986) and likely also νLa.max. It has been shown, that a reduction of carbohydrate intake combined with physical exercise for 2 days led to the depletion of muscle glycogen stores (Balsom et al., 1999) and 4 days of high‐carbohydrate diet increased muscle glycogen levels significantly (Laurent et al., 2000). The modification of glycolytic substrate availability via nutritional inconsistencies and experimental modification might possibly influence exercise‐induced lactate production. Consequently, due to the big influence of the numerator in the equation used for νLa.max determination, all methodological variations that impact lactate buildup may lead to variations in νLa.max determination and hence misinterpretation. Further, due to differences in muscle recruitment and contraction dynamics νLa.max values do not correlate well between different exercise modes particularly not between running and cycling (Quittmann, Schwarz, et al., 2021). This requires detailed knowledge about influencing factors that may impact the reliability of νLa.max determination and as a consequence the requirement for specific νLa.max methodologies between different exercise modes. Additionally, multiple olympic disciplines rely on running movements and νLa.max values determined in running sprint tests (RST) are more specific for such athletes (Quittmann, Schwarz, et al., 2021). Therefore, and because νLa.max data determined through running sprints are still sparse, we chose RST as methodological basis in our intervention study. We examined the variations in νLa.max determination after running sprints when La_rest_ is altered via changing the recovery duration after warmup and carbohydrate intake is experimentally manipulated beforehand RST. We highlight here, that the individual νLa.max is usually interpreted as being lowered when resting lactate levels are increased and carbohydrate intake prior RST is reduced (Leija et al., 2024; Millard‐Stafford et al., 2010; Woerle et al., 2003).

## 2 MATERIALS AND METHODS

### 2.1 Participants

Thirteen male and eight female (*N* = 21; Table 1) sport students of the University of Hildesheim participated in this study. Body height (cm) and body mass (kg) were measured manually with a Seca roll‐up measuring tape 206 and a Seca scale Sensa 804 (SECA GmbH & Co KG; Hamburg, Germany). Body fat and muscle mass were determined using a Medical Body Composition Analyzer (SECA mBCA 525) (SECA GmbH & Co KG; Hamburg, Germany). Data are shown in Table 1.

**TABLE 1:** Anthropometric data.

Parameters	Total	Male	Female
	*N* = 21	*n* = 13	*n* = 8
Age [years]	23.1 ± 1.9	23.3 ± 2.1	22.7 ± 1.6
Height [cm]	177.0 ± 8.3	181.7 ± 6.5	169.4 ± 4.4
Body mass [kg]	74.2 ± 11.8	81.1 ± 9.1	63.0 ± 5.3
Body fat [%]	20.7 ± 6.8	17.4 ± 5.7	26.1 ± 4.8
Muscle mass [kg]	28.9 ± 6.5	33.4 ± 3.5	21.6 ± 2.0

*Note*: Data are presented as arithmetic means and standard deviation (SD).

Before written consent was obtained, participants were informed about the nature and risks of the experimental procedures. This study was approved by the ethical committee of the University of Hildesheim (Nr. 301) and conformed the standards of the Declaration of Helsinki.

### 2.2 Running Sprint testing (RST)

**FIGURE 1:**
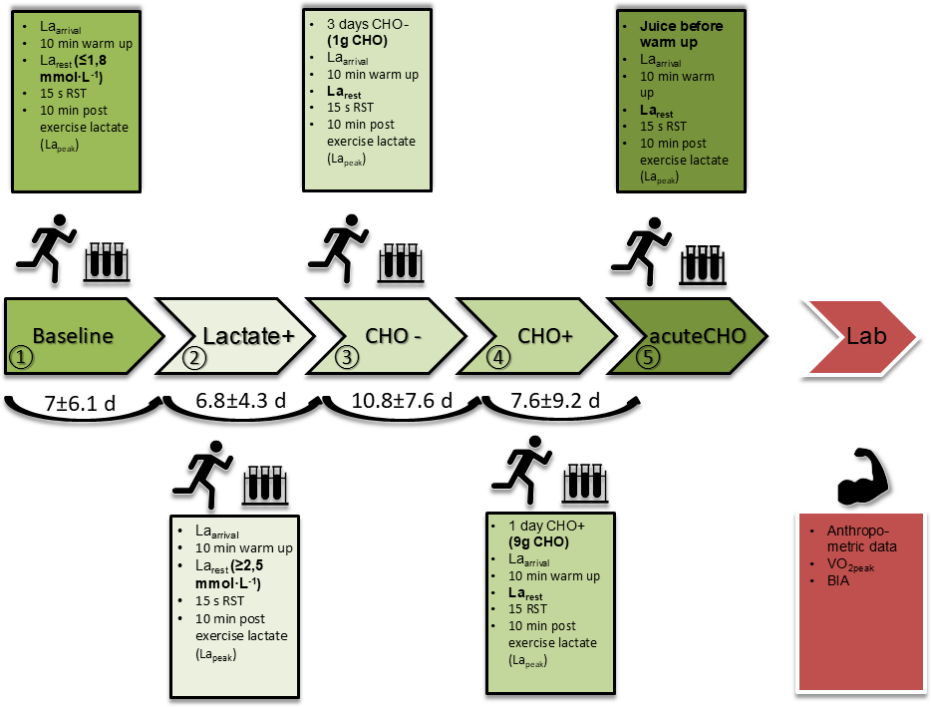
Illustration of the study timeline. At five subsequent occasions 15‐s RST (baseline‐acuteCHO) tests were conducted under varying conditions followed by subsequent laboratory measurements of blood glucose and lactate levels. The days (d) between RST are depicted as arithmetic mean and standard deviation.

The participants were required to arrive by bus or car to avoid intense physical activity beforehand. All RST were conducted in the morning between 8 am and 10 am in a fasted and rested state in the order depicted in Figure 1.

Five conditions were performed in the specified order on separate days by each participant: First, baseline RST was conducted, followed by lactate+, CHO−, CHO+, and finally acuteCHO. Properties of the specific testing conditions in CHO−, CHO+ and acuteCHO are described in detail in chapter 2.6.

The participants did not perform any exercise within at least 24 h prior to the RST. After arrival at the running track, initial blood lactate (La_arrival_) and glucose (Glu_arrival_) samples were obtained from the participants in a sitting position. Then, the RST started with a standardized 10‐min warmup that included mobilization, activation of the muscles of the lower limbs and acceleration runs to prepare for a following maximal sprint. The specific conditions of all warmup exercises are displayed in Table 2.

**TABLE 2:** Standardized warmup. Sequence of exercises with distance covered and intensity.

Block	Exercise	Distance	Intensity
Mobilization	Single knee tuck	8 m	Low
	Hamstring stretch	8 m	Low
	Quad stretch	8 m	Low
	Static external hip rotation	8 m	Low
	Side lunges right	8 m	Low
	Side lunges left	8 m	Low
	Lunge and upper body rotation	8 m	Low
Activation	Moderate jog	8 m	Moderate
	High‐knee running (right)	8 m	Moderate
	High‐knee running (left)	8 m	Moderate
	Kick backs	8 m	Moderate
	Hamstring pulls	8 m	Moderate
	Wide hamstring pull	8 m	Moderate
	Skippings	8 m	Low
	Skippings	8 m	Moderate
Acceleration	Acceleration run to 80% *v*_max_	24 m	High
	Acceleration run to 90% *v*_max_	24 m	High
	Acceleration run to 100% *v*_max_	24 m	Maximal

After performing each exercise for one time (Table 2, column *Exercise*), the participants returned the covered distance (Table 2, column *Distance*) to the start with a low intense run and continued immediately with the next exercise. Each exercise was demonstrated by the investigator. Intensities (Table 2, column *Intensity*) were instructed by the investigator and applied individually by the participants. After the warmup, participants followed a time of walking and sitting until the desired La_rest_ of the particular condition (at baseline and lactate+) was reached. In the baseline RST, participants attained La_rest_ of ≤1.5 mmol·L^−1^, in lactate+ La_rest_ of ≥2.5 mmol·L^−1^ was attained. The participants only remained seated during the lactate+ RST procedure immediately after the warmup and did not have a predetermined walking time as in the other RSTs. In CHO−, CHO+ and acuteCHO RST, the participants followed the same walking and sitting procedure as in baseline.

When the warmup was performed and the La_rest_ criteria was met (in baseline and lactate+), or the walking and sitting procedure was finished (in CHO−, CHO+ and acuteCHO), participants performed a 15‐s RST on a running track. The duration of RST was determined because it has been shown that glycolytic activity reaches its peak at 15‐s (Walter et al., 1999). A signal horn determined the start and the end of the sprint. Immediately after the test, participants went into a seating position. For the determination of La_peak_, capillary blood was obtained from the earlobe in one‐minute intervals over a total time of 10‐min after the sprint. The lactate values were also needed to calculate the νLa.max.

The formula of νLa.max is depicted in Equation 1:

(1) $$\nu_{La.\max}(\text{mmol}\cdot {\text{L}}^{-1}\cdot {\text{s}}^{-1})=\frac{La_{\text{peak}}-La_{\text{rest}}}{t_{\text{Exer}}-t_{PCr}}$$

La_peak_ = the maximum lactate concentration after 15‐s RST; La_rest_ = the resting lactate concentration (within 20‐s) before 15‐s RST; *t*_Exer_ = total exercise (running) time (15‐s) and *t*_PCr_ = the time of energy metabolism relying on ATP and PCR (Heck et al., 2003).

The alactic time of the test in which the dominant phosphagen‐contributed time (the energy turnover is mainly dependent on ATP and PCR) (Gastin et al., 1995; Hargreaves & Spriet, 2020; Heck et al., 2022; Yang et al., 2023) was calculated using an interpolated model that has been reported previously (Quittmann et al., 2020) (Equation (2)):

(2) $$t_{\mathrm{PCr}}\;\left(s\right)=t_{\text{Exer}}\cdot 0.0909+2.0455$$

In our case, a value of 3.4‐s was used for *t*_PCr_, which is the result when inserting 15‐s for *t*_Exer_ in Equation 2. This time was used to calculate νLa.max for all participants and all RST conditions.

### 2.3 Lactate and glucose diagnostics

For the determination of lactate/glucose concentration, capillary blood samples (mmol·L^−1^; 20 μL) were collected from the earlobe into an end‐to‐end capillary (EKF diagnostic, Barleben, Germany). Immediately after collection, the blood from the capillary was mixed with a hemolyzing solution. An enzymatic‐amperometric sensor (Biosen C‐line, EKF diagnostics sales GmbH, Barleben, Germany) was used for analysis of all samples. La_rest_ and resting glucose (Glu_rest_) samples were analyzed immediately after collection to control for desired La_rest_ conditions in baseline and in lactate+, whereas La_arrival_, Glu_arrival_ and post‐exercise samples were analyzed within 60‐min after the sprint. Blood samples in lactate+ were collected subsequently one after another until the lactate value was ≥2.5 mmol·L^−1^. For Glu_arrival_ and Glu_rest_, 11 samples were analyzed in CHO− and CHO+ conditions, whereas baseline and lactate+ samples were not. In acuteCHO, 19 samples were analyzed for Glu_arrival_ and Glu_rest_. Instead of the missing Glu_arrival_ values in baseline, we used the values from acuteCHO. These conditions were identical until the point in time of arrival. An automatic calibration of the system was performed after 60‐min.

### 2.4 Lactate and glucose test in whole blood, erythrocytes, and plasma

To test whether acute glucose ingestion will affect glucose uptake and lactate production by erythrocytes already without exercise, we conducted a differential lactate analysis in a small subset of athletes. Determination of lactate and glucose concentration in whole blood, erythrocytes, and blood plasma was performed under fasting conditions subsequent to all described RST in four male judoka athletes (*N* = 4, 177.5 ± 9.7 cm, 93.5 ± 30.8 kg, 22.75 ± 3.7 years). Samples were collected before, after 3 min, and after 10 min following consumption of a glucose‐containing beverage. All whole blood samples were obtained using an end‐to‐end capillary (mmol·L^−1^; 20 μL). Immediately after collection, the blood from the capillary was mixed with a hemolyzing solution and evaluated using an enzymatic‐amperometric sensor chip system (Biosen C‐line, EKF diagnostics sales GmbH, Barleben, Germany). Hematocrit samples were also collected using an end‐to‐end capillary (mmol·L^−1^, 50 μL, Hirschmann, Eberstadt, Germany). Immediately after blood collection, samples of cell fractions containing erythrocytes were sealed (Brand, Wertheim, Germany) and centrifuged for 2 min at 13,000 rpm (Hettich Hematocrit 210, Tuttlingen, Germany). Subsequently, cell fractions containing erythrocytes and plasma components were separated, and 20 μL of each fraction were pipetted (EP Research Plus G, single‐channel pipette, Eppendorf, Hamburg, Germany) and mixed with a hemolyzing solution and analyzed with the sensor chip system. The values of hematocrit and plasma, each totaling 20 μL, were divided by two to account for the approximate 50/50 distribution of both fractions within whole blood. The hematocrit value is a percentage of the erythrocyte mass in the human body and is therefore represented as “Erythrocytes” in Figure S1 (Gaillard & Hamilton, 1986).

### 2.5 V̇O_2peak_ testing

Peak oxygen uptake (V̇O_2peak_) was measured using a metabolic analyzer K5 (COSMED, Rome, Italy) in mixchamber mode (Winkert et al., 2020). Gas was collected for 10‐s intervals and then measured (mL/min) using Omnia Cardiopulmonary Diagnostic Software version 1.6.10 (COSMED, Rome, Italy). The highest 10‐s interval determined the V̇O_2peak_. Before each V̇O_2peak_ testing the flow sensor was calibrated with five strokes of a high precision 3 L syringe (Hans Rudolph, Kansas City, MO, USA). Gas calibration was performed with calibration gas (16% O_2_ and 5% CO_2_, COSMED, Rome, Italy). All athletes wore appropriate silicone masks which were attached to the flowmeter of the K5 system (Hans Rudolph, Kansas City, MO, USA).

Heart rate (bpm) was continuously measured during the V̇O_2peak_ testing using a Vitalmonitor Flow HRV (Viita Holding GmbH, Traun, Austria). The testing protocol started with a two‐minute warmup stage at 100 W for women and 150 W for men followed by an increase of 25 W every 30‐s until exhaustion (Hauser et al., 2014). Participants had to remain seated throughout the entire test and were encouraged to maintain a pedaling frequency between 80 and 90 rpm. The test was terminated when the participants were unable to continue the test due to fatigue.

### 2.6 Dietary intake conditions and analysis

A complete food diary of the previous day (Table 3) was collected on the day of baseline and lactate+ RST and analyzed with NutriGuide software to assess carbohydrate intake of all participants (NutriScience, Freiburg, Germany). Before CHO−, participants had to follow three days of a low‐carbohydrate diet (≤1 g CHO·kg ^−1^ bodyweight · d^−1^) (Burke, 2021) and were advised to record their nutritional behavior by additional food diaries. In order to reduce muscle and liver glycogen levels participants had to perform a self‐paced 60‐min endurance run with moderate intensity (rate of perceived exertion 5/10) on the first two of the three consecutive days (Goforth et al., 2003). On the third day, participants were instructed to rest in preparation for the subsequent CHO− while still consuming a reduced amount of carbohydrates. In the morning of the 4th day, the participants went to the lab to conduct RST under the CHO− condition. In CHO+, participants had to follow 1 day of a high‐carbohydrate diet (≥9 g·kg^−1^ bodyweight · d^−1^) (de Moraes et al., 2019) and were advised to record their nutritional intake via an additional food diary. Carbohydrate consumption levels for all conditions are depicted in Table 3. For the acuteCHO RST, participants consumed immediately prior to the warmup of the RST (5‐min approximately) 500 mL of a glucose‐containing beverage (Rewe Beste Wahl, Cologne, Germany) containing 36 grams carbohydrates of which 34 grams were sugar.

**TABLE 3:** Blood lactate/glucose levels and determined carbohydrate intake before RST (baseline, lactate+, CHO+, and acuteCHO—assessment of the last 24 h; CHO−assessment of the last 72 h.

Parameters	Baseline	Lactate+	CHO−	CHO+	acuteCHO
CHO intake (g · kg^−1^ bodymass · d^−1^)	3.38 ± 1.61 (*N* = 21)	3.67 ± 1.63 (*N* = 21)	0.86 ± 0.41 (N = 21)	8.21 ± 2.37 (N = 21)	3.98 ± 1.68 (*N* = 21)
La_arrival_ (mmol · L^−1^)	0,78 ± 0.36 (*N* = 21)	0,7 ± 0.36 (*N* = 21)	0.58 ± 0.14 ** (*N* = 21)	0.84 ± 0.27 (*N* = 21)	0.74 ± 0.19 (*N* = 21)
La_rest_ (mmol · L^−1^)	1.37 ± 0.33 (*N* = 21)	3,37 ± 0.54 (*N* = 21)	1.18 ± 0.4 (*N* = 21)	1.56 ± 0.66 (*N* = 21)	2.2 ± 0.86 (*N* = 21)
La_peak_ (mmol · L^−1^)	8.28 ± 1.17 (N = 21)	9.37 ± 1.32 (N = 21)	7.33 ± 1.33 (N = 21)	7.89 ± 1.33 (N = 21)	8.9 ± 1.31 (*N* = 21)
Glu_arrival_ (mmol · L^−1^)	/	/	3.97 ± 0.27 (*n* = 11)	4.31 ± 0.24 (n = 11)	4.3 ± 0.19 (*n* = 11)
Glu_rest_ (mmol · L^−1^)	/	/	4.34 ± 0.27 (n = 11)	4.53 ± 0.29 (n = 11)	5.93 ± 1.04 (*n* = 11)
νLa.max (mmol · L^−1^ · s^−1^)	0.59 ± 0.09 (*N* = 21)	0.51 ± 0.01 (N = 21)	0.53 ± 0.1 (N = 21)	0.54 ± 0.1 (N = 21)	0.57 ± 0.1 (*N* = 21)
∆ La_peak_‐La_rest_ (mmol · L^−1^)	6.91 ± 1.06 (N = 21)	5.99 ± 1.26 (N = 21)	6.15 ± 1.19 (N = 21)	6.32 ± 1.23 (N = 21)	6.67 ± 1.25 (*N* = 21)
∆ Glu_rest_ – Glu_arrival_ (mmol · L^−1^)	/	/	0.36 ± 0.31 (n = 11)	0.19 ± 0.19 ** (*n* = 19)	1.53 ± 0.89 (*n* = 19)
∆ La_rest_ – La_arrival_ (mmol · L^−1^)	0.59 ± 0.49 (N = 21)	2.67 ± 0.58 (N = 21)	0.6 ± 0.38 (N = 21)	0.72 ± 0.59 ** (N = 21)	1.48 ± 0.73 (*N* = 21)

*Note*: Data are presented as aritmetic mean and standard deviation.

### 2.7 Statistical analysis

All data were analyzed using GraphPad Prism (version 8.0.2, GraphPad Prism Software Inc., La, Jolla, CA, USA) and IBM SPSS Statistics (version 28.0, SPSS IBM, Stamford, USA). Parameters are presented as arithmetic mean and standard deviation (SD). The arithmetic mean delta values (∆) are additionally provided. The data were assessed for normal distribution with the shapiro–wilk‐test. The Friedman multiple‐comparisons with Dunn's post hoc test was utilized to compare the individual La_rest_, La_arrival_, La_peak_, νLa.max and carbohydrate intake values for the different points in time (baseline, lactate+, CHO−, CHO+, acute CHO). A one‐way analysis of variance (ANOVA) with the uncorrected Fisher's LSD was utilized for the Glu_rest_ and Glu_arrival_ values in CHO−, CHO+ and acuteCHO diagnostics. To determine the differences between Glu_arrival_ and Glu_rest_ in acuteCHO, a paired *t*‐test was conducted. For comparing the unequal sample sizes, the Mann–Whitney *U* test was employed. The α‐level of significance was set at *p* < 0.05 for all statistical analyses. The effect sizes were calculated for nonparametric tests ($\frac{Z}{\sqrt{N}}$ [r]). The thresholds for small, medium, and large effects were considered 0.1, 0.3, and 0.5 [*r*]. The Cohen's d [*d*] was calculated for parametric tests. The thresholds for small, medium, and large effects were 0.2, 0.5, and 0.8 for Cohen's d [*d*] (Fritz et al., 2012). The relationship between Glu_rest_ vs La_rest_ in acuteCHO and ∆Glu_rest‐_Glu_arrival_acuteCHO vs ∆La_rest‐_La_arrival_acuteCHO was analyzed with a two‐tailed Pearson's correlation. The Spearman correlation was employed to calculate the relationship between νLa.max and V̇O_2peak_ at baseline. The significance level was set at *p* < 0.05.

## 3 RESULTS

All determined parameters within the study as well as the carbohydrate intake for all conditions are presented in Table 3.

**FIGURE 2:**
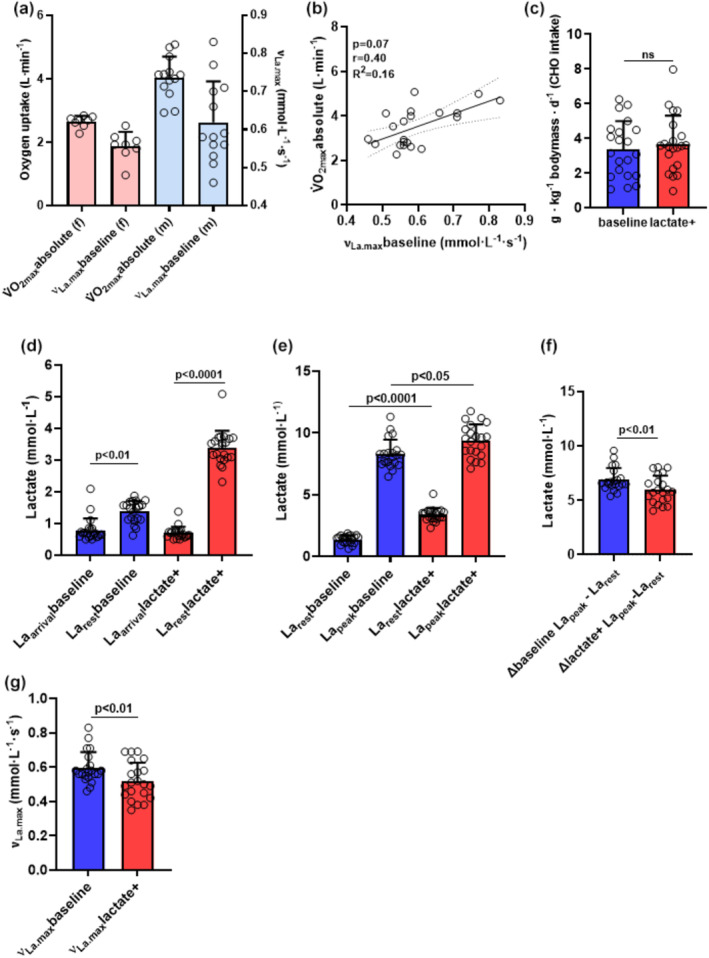
Illustration of the effects of increased La_rest_ levels on La_peak_ and νLa.max. The depicted values represent the arithmetic means ± standard deviations (SD) and individual data points for all participants from a 15‐s RST in baseline (blue bars) and lactate+ (red bars) conditions (*N* = 21). (a) Absolute oxygen uptake (V̇O_2peak_, L·min^−1^) for women (*n* = 8, light red bar) and men (*n* = 13, light blue bar). (b) two tailed spearman correlation between V̇O_2peak_ and νLa.max (mmol·L^−1^·s^−1^). Dotted lines: 95% confidence interval, straight line: Strength of the linear relationship between two variables. (c) Carbohydrate intake (in g · kg^−1^ body mass · d^−1^) on the day prior to the 15‐s RST. (d) Resting lactate levels (mmol· L^−1^) immediately upon the participants' arrival (La_arrival_) and the lactate levels following the warmup (La_rest_). (e) La_rest_ and La_peak_ values (mmol· L^−1^). (f) ∆ values of La_peak_ and La_rest_ (net lactate accumulation, mmol· L^−1^). (g) maximal glycolytic rate (νLa.max, mmol·L^−1^·s^−1^).

Baseline aerobic capacity (V̇O_2peak_) differed significantly between females 2.61 ± 0.15 and males 4.03 ± 0.53 L·min^−1^ (Figure 2a). Baseline νLa.max was 0.59 ± 0.09 mmol · L^−1^ · s^−1^ and correlated moderately with V̇O_2peak_ within the entire group (r = 0.40, *p* = 0.07, Figure 2b).

### 3.1 Effects of increased La_rest_ levels on La_peak_ and νLa.max

We investigated whether and how increased La_rest_ values will affect La_peak_ and νLa.max after RST. To assess the initial nutritional behavior of the participants we analyzed nutritional diaries the day/s before RST. There were no significant differences observed in carbohydrate intake the day before baseline and lactate+ RST (Figure 2c; Table 3).

Acutely before conducting the RST in baseline and lactate+, La_rest_ levels after warmup were significantly higher compared to the resting lactate levels immediately upon arrival (La_arrival_) (*p* < 0.01, *ES*[*r*]:*0*.*72* and *p* < 0.0001, *ES*[*r*]: *0*.*87*, Figure 2d; Table 3). This indicates that the warmup procedures already required the involvement of skeletal muscle glycolysis and hence lactate production. After warmup, lactate levels reached in some participants up to ≥3.7 mmol· L^−1^ (data not shown). As specifically aimed, La_rest_ levels were higher in the lactate+ compared to baseline condition (*p* < 0.0001, *ES*[*r*]: *0.87*, Figure 2e; Table 3). Correspondingly, La_peak_ levels were significantly higher in lactate+ than in baseline (*p* = 0.012, *ES*[*r*]: *0*.77, Figure 2e; Table 3). Surprisingly, the difference between La_rest_ and La_peak_ was significantly reduced in lactate+ (*p* < 0.01, *ES*[*r*]: *0*.*70*, Figure 2f; Table 3) compared to baseline. This suggests that elevated La_rest_ levels blunt the further post RST accumulation and prevent a similar net lactate accumulation as observed with lower La_rest_ (≤1.5 mmol L^−1^) in baseline. Consequently νLa.max was significantly lower in lactate+ RST compared to baseline (*p* < 0.01, *ES*[*r*]: *0*.*68*, Figure 2g; Table 3).

### 3.2 Effects of low and high carbohydrate intake on blood glucose levels, La_rest_, La_peak,_ and νLa.max

**FIGURE 3:**
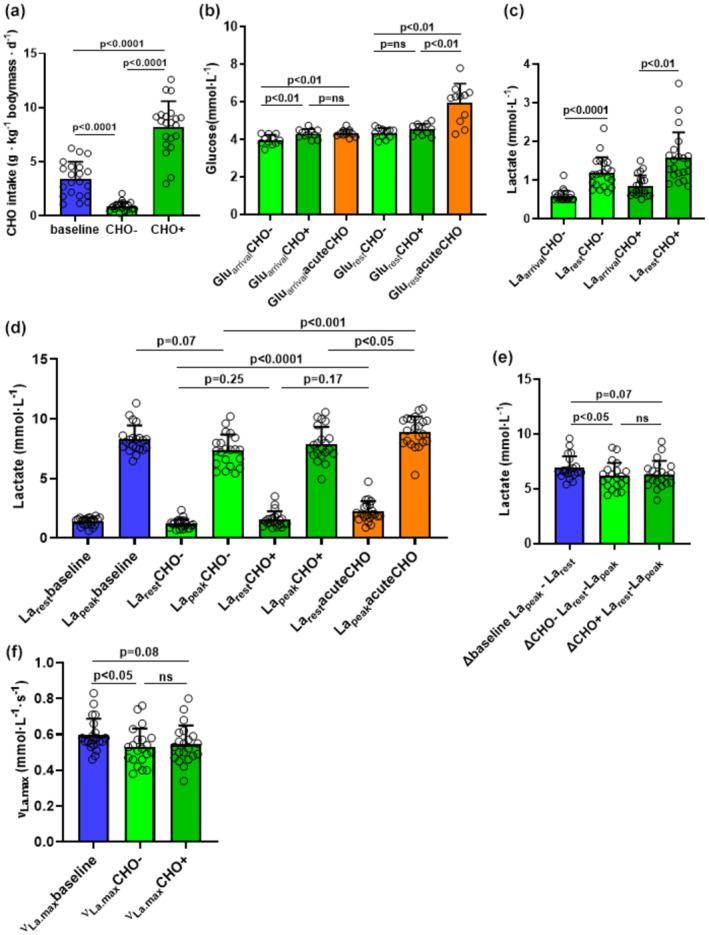
Illustration of effects of low and high carbohydrate intake on blood glucose levels, La_rest_, La_peak_ and νLa.max. The depicted values represent the arithmetic means ± standard deviations (SD) and individual data points for all participants from a 15‐s RST in baseline (blue bars), CHO− (light green bars), CHO+ (dark green bars) and acuteCHO (orange bars) conditions (*N* = 21 in a,c,d,e,f and *n* = 11 in b). (a) Carbohydrate intake (in g · kg^−1^ bodymass · d^−1^) on the day(s) prior to the 15‐s RST. (b) Resting glucose levels (mmol· L^−1^) immediately upon the participants' arrival (Glu_arrival_) and after warmup (Glu_rest_). (c) Resting lactate levels (mmol· L^−1^) immediately upon the participants' arrival (La_arrival_) and the lactate levels following the warmup (La_rest_). (d) La_rest_ and La_peak_ (mmol· L^−1^) values. (e) ∆ values of La_peak_ and La_rest_ (net lactate accumulation, mmol· L^−1^). (f) maximal glycolytic rate (νLa.max, mmol·L^−1^·s^−1^).

Our aim was to determine the effects of a three‐day low‐carbohydrate diet and a one‐day high‐carbohydrate diet on blood glucose levels, La_rest_, La_peak_ and νLa.max. Participants had a significantly lower carbohydrate intake in the 3 days preceding the CHO− compared to baseline (*p* < 0.0001, ES[r]: 0.87, Figure 3a, Table 3). As intended, carbohydrate intake on the day before the CHO+ RST was significantly higher compared to both baseline and CHO− (both *p* < 0.0001 and ES[r]: 0.87, Figure 3a; Table 3).

We next determined whether reduced and increased carbohydrate uptake in CHO− and CHO+ conditions affect blood glucose levels. Unfortunately, the Glu_arrival_ levels from baseline are missing. Instead, the values from acuteCHO are utilized (Figure 3b; Table 3). This is considered suitable as both conditions (baseline and acuteCHO) were identical until arrival and not affected by variations in carbohydrate uptake. Glu_arrival_ was significantly lower in CHO− compared to the acuteCHO conditions (*p* < 0.01, ES[d]: 1.3, Figure 3b; Table 3). Similarly, Glu_arrival_ was significantly reduced in CHO− compared to CHO+ (*p* < 0.01, *ES*[*d*]: 1.2, Figure 3b; Table 3). No significant differences were determined between CHO+ and acuteCHO (Figure 3b; Table 3). Thus, our data suggest that low carbohydrate intake in combination with carbohydrate depletion reduces fasting blood glucose levels whereas high carbohydrate intake levels, at least in healthy young participants, are downregulated to physiological levels after overnight fasting (Knapik et al., 1985). Also, in CHO− and CHO+ La_rest_ levels were significantly increased after warmup compared to La_arrival_ (*p* < 0.0001, *ES*[*r*]: *0*.*87* and *p* < 0.01, *ES*[*r*]: *0*.*87*, Figure 3c; Table 3). La_rest_ levels after warmup did not differ between baseline and CHO−, baseline and CHO+, as well as CHO− and CHO+ conditions (Figure 3d; Table 3). Further, La_peak_ levels after RST did not differ significantly between baseline and CHO+ (Figure 3d; Table 3). However, we determined a clear reduction of La_peak_ in CHO− compared to baseline (*p* = 0.07, *ES*[*r*]: 0.57, Figure 3d; Table 3) denoting that a reduction in glycogen content and low carb nutrition will likely reduce skeletal muscle glycolysis. Consequently, the mean ∆ values of La_peak_ to La_rest_ were significantly increased under baseline conditions compared to CHO− (*p* = 0.017, *ES*[*r*]: 0.60, Figure 3e; Table 3). A clear decrease from baseline to CHO+ was observed (*p* = 0.07, *ES*[*r*]: *0*.49, Figure 3e; Table 3). No significant differences were found between CHO− and CHO+ (Figure 3e; Table 3). As a result, νLa.max was significantly decreased in CHO− compared to baseline (*p* = 0.014, *ES*[*r*]: *0.59*, Figure 3f, Table 3) and was clearly reduced between baseline and CHO+ (*p* = 0.08, *ES*[*r*]: *0*.*49*, Figure 3f; Table 3). No differences were observed between CHO− and CHO+ conditions in νLa.max (Figure 3f; Table 3).

### 3.3 Effects of acute glucose intake prior RST on glucose levels and νLa.max

**FIGURE 4:**
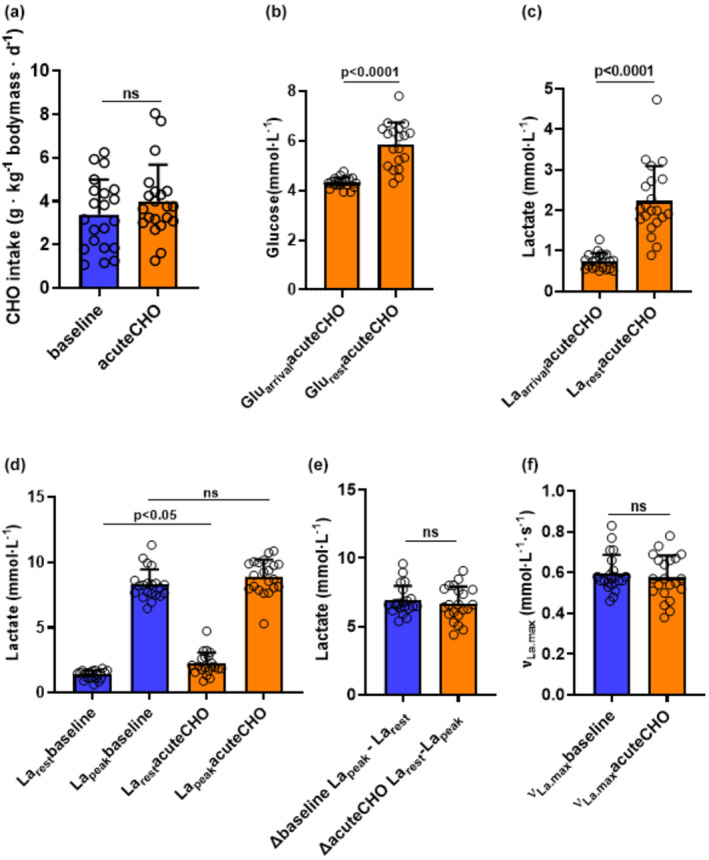
Illustration of effects of acute glucose intake prior RST on glucose levels and νLa.max. The depicted values represent the arithmetic means ± standard deviations (SD) and individual data points for all participants from a 15‐s RST in baseline and acuteCHO (orange bars) (*N* = 21 in A, C, D, E, F and *n* = 19 in B). (a) Carbohydrate intake (g · kg^−1^ bodymass · d^−1^) on the day prior to the 15‐s RST. (b) Resting glucose levels (mmol·L^−1^) immediately upon the participants' arrival (Glu_arrival_) and after warmup (Glu_rest_). (c) Resting lactate levels (mmol·L^−1^) immediately upon the participants' arrival (La_arrival_) and the lactate levels following the warmup and the glucose containing beverage (La_rest_). (d) La_rest_ and La_peak_ values (mmol·L^−1^). (e) ∆ values of La_peak_ and La_rest_ (net lactate accumulation, mmol·L^−1^). (f) maximal glycolytic rate (νLa.max, mmol·L^−1^·s^−1^).

We aimed to determine whether increases of blood glucose via acute sugar administration will affect νLa.max. On the day of acuteCHO, participants arrived also in the fasted state and consumed a glucose‐containing beverage immediately before warmup. The quantity of consumed carbohydrates did not differ between baseline and acuteCHO conditions (Figure 4a, Table 3).

Glu_rest_ was significantly increased compared to Glu_arrival_ (*p* < 0.0001, *ES*[*d*]:1.6, Figure 4b; Table 3) which likely denotes an effect of the consumed beverage. For Glu_rest_, a significant increase from CHO− to acuteCHO (*p* < 0.01, *ES*[*d*]: 0.58, Figure 3b; Table 3) as well as from CHO+ to acuteCHO was observed (*p* < 0.01, *ES*[*d*]: 0.46, Figure 2b; Table 3). Consistently with the other RST conditions, we detected also in acuteCHO a significant increase from La_arrival_ to La_rest_ levels (*p* < 0.0001, *ES*[*r*]: *0*.*87*, Figure 4c; Table 3). However, La_rest_ levels were significantly higher in acuteCHO compared to baseline (*p* = 0.012, *ES*[*r*]: 0.79, Figure 4d; Table 3) indicating that despite the same warmup procedure in all conditions, the acute sugar administration may have increased skeletal muscle glycolytic activity. Interestingly, glucose administration alone and without warmup exercises resulted in an increase in glucose and lactate levels within the erythrocyte‐containing blood fraction (Figure S1A,B). This indicates that erythrocytes contribute at least in part to the rise in lactate levels under resting conditions (Kuchel et al., 1984). We also observed a significant increase in CHO− to acuteCHO in La_rest_ (*p* < 0.0001, *ES*[*r*]: *0*.*86*, Figure 3d; Table 3). Additionally, La_rest_ levels showed a clear tendency for being higher in acuteCHO (2.2 ± 0.86 mmol · L^−1^) vs. CHO+ condition (1.56 ± 0.66 mmol L^−1^) (*p* = 0.17, *ES*[*r*]: *0*.*71*, Figure 3d; Table 3) with few participants accounting for the high variability in CHO+ and acuteCHO. La_peak_ levels were not different between baseline and acuteCHO, but significantly increased after acuteCHO compared to CHO+ (*p* = 0.02, *ES*[*r*]: *0.79*, Figure 3d; Table 3). We also observed a significant increase in La_peak_ from CHO− to acuteCHO (*p* < 0.001, *ES*[*r*]: *0*.*77*, Figure 3d; Table 3). In line with that, ∆ values of net lactate production were not different between the baseline and acuteCHO condition (Figure 4e; Table 3). Also νLa.max was not different between acuteCHO and baseline (Figure 4f; Table 3).

**FIGURE 5:**
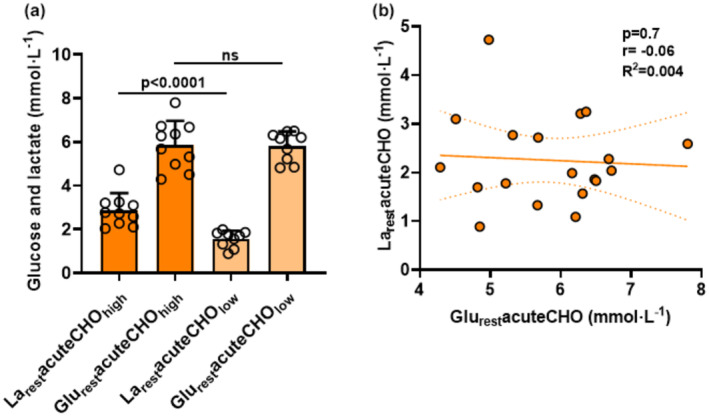
(a) Illustration of differences between the two groups La_rest_acuteCHO_high_ (≥1.9 mmol · L^−1^) and Glu_rest_acuteCHO_high_ (orange bars, *n* = 10) vs La_rest_acuteCHO_low_ (≤1.9 mmol · L^−1^) and Glu_rest_acuteCHO_low_ (light orange bars, *n* = 9). The depicted values represent the arithmetic means ± standard deviations (SD) and individual data points for all participants from a 15‐s RST in acuteCHO. (b) Correlation between La_rest_acuteCHO and Glu_rest_acuteCHO (mmol· L^−1^) (*n* = 19). Dotted lines: 95% confidence interval, straight line: Strength of the linear relationship between two variables.

In order to determine whether glucose levels after glucose ingestion and warmup correlate with the increase in resting lactate before RST, we separated the participants in two groups differentiating in _low_ (≤1.9 mmol L^−1^) and _high_ resting lactate (≥1.9 mmol L‐1) (Figure 5a). Absolute glucose and resting lactate levels after warmup did not correlate in these groups (*high: p* = 0.4, *r* = 0.26, *R* ^2^ = 0.07, *low: p* = 0.4, *r* = 0.36, *R* ^2^ = 0.13). The significantly higher increase in lactate levels (*p* < 0.0001, La_rest_acuteCHO_high_: 2.88 ± 0.78 mmol· L^−1^, La_rest_acuteCHO_low_: 1.56 ± 0.37 mmol· L^−1^) was not reflected in the glucose levels (*p* > 0.05, Glu_rest_acuteCHO_high_: 5.86 ± 1.10 mmol· L^−1^, Glu_rest_acuteCHO_low_: 5.80 ± 0.68 mmol· L^−1^) between those two groups (Figure 5a). This indicates that increased blood glucose levels had no causal effect on increased La_rest_ levels in our study (*p* > 0.5, *r* = −0.06, *R* ^2^ = 0.004, Figure 5b).

## 4 DISCUSSION

In our study we aimed to detect the effect of (1) increased resting lactate levels, (2) reduced and (3) increased carbohydrate intake as well as (4) acute sugar consumption on lactate accumulation and νLa.max after a 15‐s maximal RST. To our knowledge, yet no other study explicitly covered such questions on a practically relevant issue in νLa.max performance diagnostics. In our participants we determined a bandwidth of νLa.max between 0.46 and 0.77 mmol · L^−1^ · s^−1^ which is in the mid‐range for νLa.max data determined in sports, higher than in elite endurance athletes but lower than in elite sprinters (Wackerhage et al., 2022). Hence, we anticipate that our results can be transferred at least to the general population of athletes ranging within such νLa.max levels.

We highlight three key results: Firstly, we demonstrate that net blood lactate accumulation is blunted when resting lactate levels are elevated above 2.5 mmol · L^−1^ (3.37 ± 0.54 mmol · L^−1^). Secondly, we determined that a low carb diet for 72 h in combination with an endurance exercise‐induced reduction of intramuscular glycogen reduces La_peak_ and by that net lactate accumulation and νLa.max. Thirdly, we show that although acute sugar administration before an RST significantly increases resting lactate, also post‐RST La_peak_ is elevated which results in an unchanged νLa.max compared to baseline RST conditions. We separated the RSTs by in mean of at least of 6.8 days, as Heldt et al. demonstrated that high reliability of νLa.max is achieved when νLa.max tests are conducted on two different days 1 week apart (ICC = 0.85) (Held et al., 2024). We explicitly compared RST‐derived νLa.max measurements five times and using the same test protocol while our observations were only dependent on the specific conditions applied before RST. In our study, all RST procedures were conducted after an overnight fasting and after a standardized warmup procedure which consistently increased lactate levels between arrival and before RST in all groups (Figures 2d, 3c, and 4c). Methodologically, we were able to exactly modulate La_rest_ levels at lactate+ by varying individual sitting durations following a standardized and equal warmup procedure (Figure 2d).

It is intriguing why La_peak_ is not elevated correspondingly to the difference between resting lactate 1.37 ± 0.33 mmol·L^−1^ at baseline and 3.37 ± 0.54 mmol·L^−1^ at lactate + (Figure 2e).

We must assume that skeletal muscle and blood lactate buffering capacity (Saltin et al., 1995) as well as glycolytic activity will not have been changed coincidentally only at lactate+ RST. However, it might be speculated that within the shorter resting phase between warmup and RST at lactate+ warmup‐induced reductions in intramuscular pH levels remained reduced at the onset of RST, relieving an inhibitory effect on the glycolytic key enzyme phosphofructokinase (PFK) (Dobson et al., 1986; Trivedi & Danforth, 1966).

Our data further introduce a reduction in La_peak_, net lactate accumulation and νLa.max after a three‐day low‐carb diet in combination with endurance exercise likely influenced by reduced glycogen levels reducing glycolytic activity and lactate production in skeletal muscle (Jacobs, 1981). Due to methodological limitations, we were not able to determine muscle glycogen levels why we can only speculate about this reduction. However, as participants reported for all RST procedures to the lab in the fasted state and glucose and lactate levels were significantly reduced in CHO− (Figure 3b) compared to all other conditions, we assume that substrate stores were at least partially reduced (Areta & Hopkins, 2018). Indeed, decreased lactate formation under resting conditions after a 5 week low‐carb diet (Hu et al., 2020) and during exercise (Jacobs, 1981) has been determined previously.

The rapid decrease of the resting respiratory exchange ratio (RER) during a low‐carb diet (Peters et al., 2001; Sparks et al., 2006) and the decreased glucose disposal after a low‐carb diet is in part related to decreased oxidative carbohydrate disposal in skeletal muscle and not decreased glycogen stores (Pehleman et al., 2005). This indicates that the mechanisms of lactate production are not only dependent on substrate availability but also on enzymatic activity. An important glycolytic enzyme is lactate dehydrogenase (LDH), which converts pyruvate and NADH to lactate and NAD + (Farhana & Lappin, 2024). It has been shown that LDH activity was lower at rest and during particular stages of an incremental exercise protocol after 4 weeks of a low‐carb diet in cyclists (Zajac et al., 2014). Further, also the activity of the rate limiting glycolytic enzyme PFK is decreased after a 175‐day low‐carb diet in rats causing reduced glycolytic flux rates resulting from the low‐carb diet (Clark et al., 1982). Those data point to a role for a nutrition‐induced temporal reduction in glycolytic enzymes in our study and as a further explanation for the decreased νLa.max in CHO−. Intriguingly La_peak_ was not increased as a result of a one‐day high‐carb diet at CHO+. This was surprising, as we expected that the enhancement of substrate availability compared to CHO‐ may change conditions rapidly to the opposite. It has been shown that a five‐day “high‐fat low‐carb” diet blunts glycolytic energy metabolism even after a subsequent 1 day high‐carb diet (Burgomaster et al., 2008; Burke et al., 2002; Helge & Kiens, 1997).To prevent adaptational interactions between two subsequent RST conditions we separated CHO− and CHO+ RST by in mean by 10.8 ± 7 d. Those findings indicate a yet undefined but extended time frame of a low‐carb diet blunting the glycolytic energy metabolism in skeletal muscle and might explain, why La_rest_ as well as La_peak_ were not higher in CHO+ compared to CHO−. On the other hand, La_peak_ levels and νLa.max were also not different between CHO+ and baseline. Therefore, a high‐carb diet which is applied regularly will not necessarily increase substrate stores and glycolytic activity when there is no substantial preempting of glycogen stores (Bergström & Hultman, 1966; Hearris et al., 2018). It can therefore not be excluded that the CHO+ condition would have created the similar effect when compared to baseline alone and without previously conducted CHO−.

In acuteCHO, blood glucose levels significantly increased when warmup was finished (Figure 4b). Importantly, at this time point, glucose levels, were also significantly higher than after overnight fasting in CHO+, thus indicating an effect induced by the administration of the glucose drink (Figure 3b). Concomitantly, despite an equal warmup procedure La_rest_ levels were also significantly higher in acuteCHO compared to baseline and CHO‐ but tendentially also higher than in CHO+ (Figure 3d). We expected that CHO+ and acuteCHO would provide similar results based on the potential effect of increased glucose availability for glycolysis. Interestingly, despite a 24‐h lasting high carbohydrate diet, blood glucose and lactate levels after warmup were not increased in CHO+ comparable to acuteCHO, where participants also arrived after an overnight fasting. Studies have shown that the consumption of a CHO drink prior to exercise led to an increase in blood lactate at early stages of a graded field test in swimmers (Millard‐Stafford et al., 2010) and after a high‐intensity intermittent running exercise (de Sousa et al., 2007). Because La_rest_ levels after warmup were higher in acuteCHO compared to CHO‐ and baseline but tendentially also higher than in CHO+, we assume three underlying mechanisms. Firstly, increasing glucose availability augments glycolysis in skeletal muscle under muscular contractions (Spriet & Watt, 2003; Wisneski et al., 1990). Secondly, the capability of red blood cells to produce lactate (Siems et al., 2000) is augmented with increasing blood glucose concentration (Kuchel et al., 1984), and thirdly the ingestion of a CHO drink enables a postprandial lactate shuttle (*PLS*) that comprises a fast enteric phase of lactate production from gut glycolysis (Leija et al., 2024). Blood lactate concentration rises from baseline within 5 min and peaks 15 min after CHO ingestion (Leija et al., 2024). Our data support these findings (Figure S1A,B) as we detected a rise of 79% in glucose and 42% in lactate levels in the erythrocyte‐containing fraction within 10 min after glucose administration and importantly without exercise. It has been also shown that 66% of ingested glucose is converted to lactate through glycolysis (Woerle et al., 2003). These findings combined might explain higher La_rest_ in acuteCHO compared to CHO+ and baseline and indicate a contribution of erythrocyte‐mediated lactate production. However, acuteCHO not only affected La_rest_ levels but also significantly increased La_peak_ levels compared to CHO‐ and CHO+ after RST (Figure 3d). Assuming that acute glucose‐induced increases in resting lactate will be maintained until Post‐RST conditions and skeletal muscle glycolytic activity will be additionally enhanced (Spriet & Watt, 2003; Wisneski et al., 1990), increased peak lactate after RST might be considered as a mixture of both events.

However, most importantly, this augmentation was responsible for maintaining the net difference between La_rest_ and La_peak_ thus also keeping νLa.max not being increased compared to CHO− and CHO+.

**FIGURE 6:**
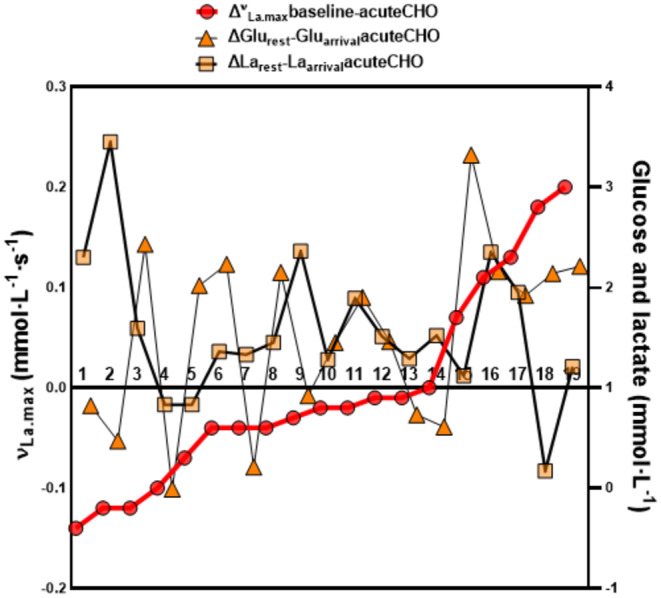
Illustration of the relationships between ∆ νLa.max (mmol·L^−1^ s^−^1) values from baseline and acute CHO compared to the ∆ lactate (mmol· L^−1^) and ∆ glucose (mmol·L^−1^) values from acuteCHO. Red dots: ∆ νLa.max from baseline and acuteCHO. Orange triangles: ∆ values from Glu_rest_ and Glu_arrival_. Light orange squares: ∆ values from La_rest_ and La_arrival_ (*n* = 19).

We recognized a high variability in the increase in blood glucose and lactate levels increase, from arrival in the fasted state to after warmup (Figure 6).

**FIGURE 7:**
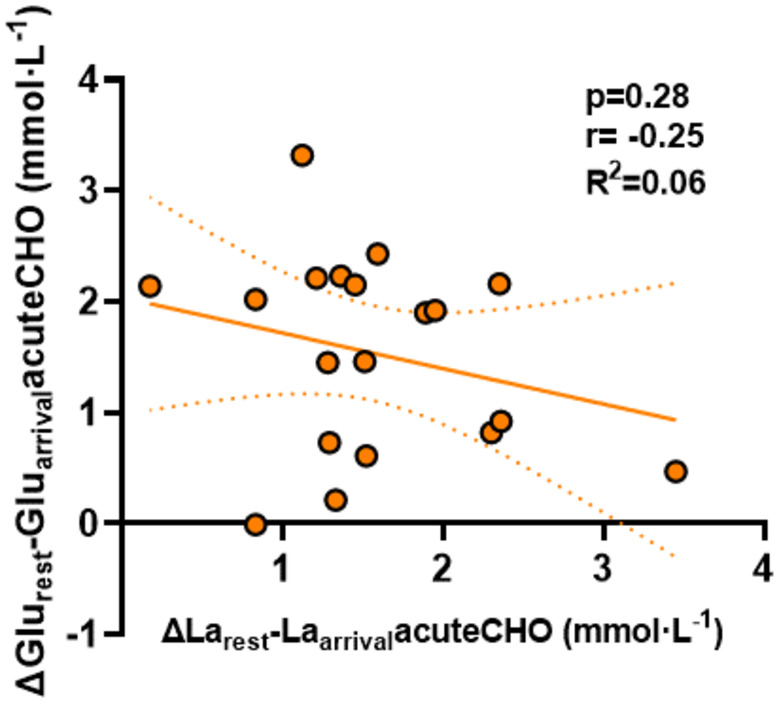
Correlation between ∆Glu_rest‐_Glu_arrival_acuteCHO and ∆La_rest‐_La_arrival_acuteCHO (mmol· L^−1^) (*n* = 19). Dotted lines: 95% confidence interval, straight line: Strength of the linear relationship between two variables.

This variability indicates that individual factors contribute to the combined response towards acute glucose consumption and exercise. It has been shown that in dependency of individual insulin responses, plasma lactate levels increase to a different extent after oral glucose administration (Berhane et al., 2015). In our study, increases in glucose and lactate (∆ levels) between arrival and after warmup did not correlate with each other (Figure 7). This emphasizes that the acute burst of glucose levels due to glucose ingestion does neither predict the magnitude of lactate increases nor the change in νLa.max from baseline.

This may suggest that especially in participants with a high increase in blood glucose levels, the majority may not be used as substrate for lactate production. Indeed, it has been shown that despite exogenous glucose administration, the majority of the produced lactate derives from intramuscular glycogen stores (Farhana & Lappin, 2024). In our case, exercise‐induced lactate production during warmup in acuteCHO may thus derive from intermuscular glycogen, a situation not distinct from baseline RST. Although in most of the participants νLa.max did not change from baseline to acuteCHO. There were some participants which either increased or decreased their individual νLa.max considerably (Figure 6, red dots). Intriguingly, there were participants with a significant increase in blood lactate (Figure 6, Light orange squares) after glucose ingestion but showing either an increase, decrease or no changes in νLa.max. This contradicts the general assumption that increased resting lactate levels reduce νLa.max. It further suggests that νLa.max determination after acute glucose ingestion is different than in lactate+ where exercise‐dependent increases in resting lactate consistently reduce the net lactate accumulation.

Despite our results we acknowledge limitations of our approach. Our procedure did not allow resting lactate levels to be exactly manipulated to a specific and predefined level. Instead, the control variable was the waiting time after the warm‐up procedure and during which lactate levels fell below the desired level. Within such a time frame, changes in blood flow or the localization of monocarboxylate transporters may have affected lactate release during the RST. Using our population of participants, we recognized an extraordinary La_rest_ response in at least within two participants (Figure 4d). Due to this variability, we were at the lower limit concerning sufficient sample size in acuteCHO. Further, we have no information concerning changes in glycolytic enzyme activities in skeletal muscle throughout the study in skeletal muscle why we can only speculate about changes at this level. Blood pH levels, blood buffering capacity and measures of insulin sensitivity (Saltin et al., 1995) at rest and after RST would have added physiological data that would have helped to get in more detail into individual variations in lactate accumulation due to RST or to acute CHO. A wider range of νLa.max in our participants would have added more information about the general transferability of our results to well‐trained athletes. It is therefore unclear, whether our data will also apply to individuals with a very low or high νLa.max. Type I and II fiber content varies considerably between healthy participants (Gehlert et al., 2012; Staron, 1997). This affects skeletal muscle lactate metabolism (Tesch et al., 1978) due to differences in glycolytic (Tesch et al., 1978) and oxidative enzymes (Gollnick et al., 1973) or monocarboxylate transporter I and IV content (Juel & Halestrap, 1999; Pilegaard et al., 1999). It can therefore be assumed that individual responses to lactate+, CHO− and acuteCHO might be regulated with different dynamics in such athletes.

Nevertheless, we determined significant differences between RST conditions in a carefully controlled training intervention. To our knowledge this is the first study to analyze these research questions in the context of νLa.max, an emerging parameter in performance diagnostics. We do not expect that the actual maximum glycolytic rate of skeletal muscle significantly changes in short time periods, as the repeated measurement of νLa.max is reliable. Hence, our data highlight that unintentional increases in resting lactate levels or unsupervised reductions in carbohydrate intake by athletes, which may easily occur in the field, lead to a too low νLa.max measurement and its underestimation. As such misinterpretations of assumed training‐induced changes may influence further decisions in training, athletes and trainers must be aware of the sensitivity of νLa.max testing. Particularly because we were not able to exactly determine the threshold at which increased resting lactate levels will affect νLa.max determination.

## 5 CONCLUSION

The consideration of standardized nutrition containing carbohydrates and resting lactate levels ≤1.5 mmol · L^−1^ is essential for reliable νLa.max testing results. Due to high interindividual differences in lactate accumulation after acute glucose intake we recommend to avoid any consumption of glucose containing beverages immediately before νLa.max determination.

## AUTHOR CONTRIBUTIONS

AP, FS and SG contributed the conception and design of the study. AP and FS obtained and analyzed the blood. The manuscript was written by AP, FS and SG. KS, OH and DJ contributed to the differential lactate diagnostics. WY contributed to statistical analysis and figure creation. All authors contributed to manuscript revision, read, and approved the submitted version.

## FUNDING INFORMATION

No funding was received for this research.

## CONFLICT OF INTEREST STATEMENT

The authors declare no conflict of interest.

## DATA AVAILABILITY STATEMENT

Data generated/analyzed during this study is available upon request from the corresponding author.

## ETHICS STATEMENT

This study was approved by the ethical committee of the University of Hildesheim and conformed the standards of the Declaration of Helsinki (Nr. 301).

## Supporting information


Figure S1.
