# Correction to “Increased resting lactate levels and reduced carbohydrate intake cause νLa.max underestimation by reducing net lactate accumulation–A pilot study in young adults”

**DOI:** 10.14814/phy2.70054

**Published:** 2024-09-16

Alexander Pohl, Frederik Schünemann, Kirill Schaaf, Woo‐Hwi Yang, Hermann Heck, Oliver Heine, Daniel Jacko, Sebastian Gehlert. Increased resting lactate levels and reduced carbohydrate intake cause νLa.max underestimation by reducing net lactate accumulation—A pilot study in young adults. *Physiological Reports* 2024;12:e70020.

In the published paper, in Figure 1 of the “2.2 Running sprint testing (RST)” section, the text “La_rest_ ≤ 1.8 mmol · L^‐1^” in the box above the baseline condition was incorrect. This should have read: “La_rest_ ≤ 1.5 mmol · L^‐1^” as shown correctly in the following figure. This change does not alter the conclusion of the study.

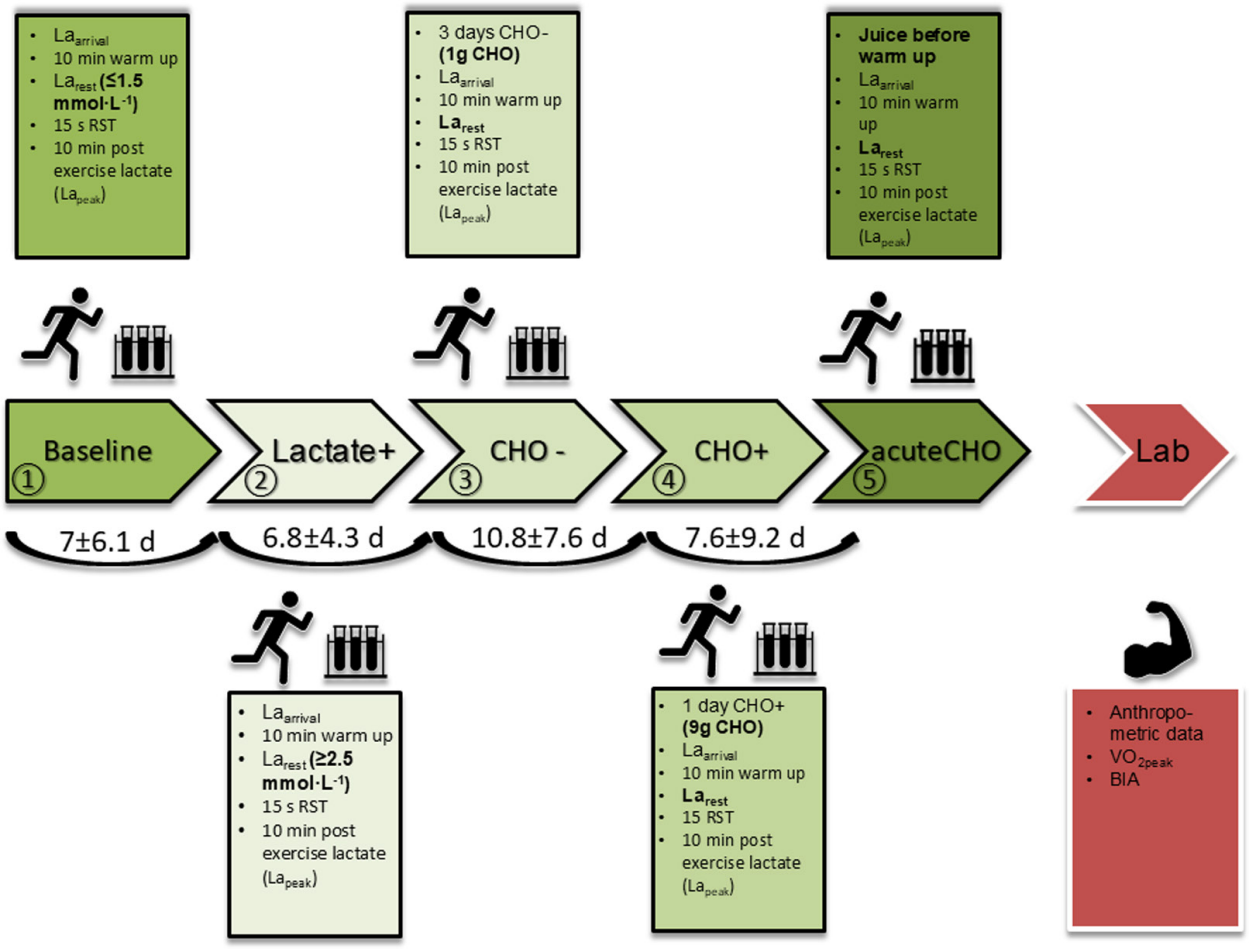

A second mistake is in paragraph 7 of the “Discussion” section. The text “Our data support these findings (Figure S1A,B) as we detected a rise of 79% in glucose and 42% in lactate levels in the erythrocyte‐containing fraction within 10 min after glucose administration and importantly without exercise.” was incorrect.

This should have read: “Our data support these findings (Figure S1A,B) as we detected a rise of 42% in glucose and 79% in lactate levels in the erythrocyte‐containing fraction within 10 min after glucose administration and importantly without exercise.” This change does not alter the conclusion of the study.

We apologize for these errors in the version of record.

