# Supplementary material for: Increased resting lactate levels and reduced carbohydrate intake cause νLa.max underestimation by reducing net lactate accumulation—A pilot study in young adults
Source: Physiol Rep. 2024 Aug 26;12(16):e70020. doi: 10.14814/phy2.70020 (PMC11347020; doi:10.14814/phy2.70020)
Supplement: Supplementary file 1 — Figure S1. [file PHY2-12-e70020-s001.rtf]

Supplementary Figure 1: Illustration of lactate and glucose levels in whole blood, plasma and erythrocytes under resting conditions before (PRE) and after (3' and 10') intake of the glucose containing beverage. The depicted values represent the means ± standard deviations (SD) and individual data points for all participants (N=4). (a) Lactate levels (mmol· L-1) in whole blood (red bars), plasma (yellow bars) and erythrocytes (blue bars) (b) Glucose levels (mmol· L-1) in whole blood, plasma and erythrocytes.
